# The DecisionDx-UM Gene Expression Profile Test Provides Risk Stratification and Individualized Patient Care in Uveal Melanoma

**DOI:** 10.1371/currents.eogt.af8ba80fc776c8f1ce8f5dc485d4a618

**Published:** 2013-04-09

**Authors:** J. William Harbour, Royce Chen

**Affiliations:** Vice Chairman for Translational Research Professor of Ophthalmology Director, Ocular Oncology Service Bascom Palmer Eye Institute University of Miami School of Medicine; Bascom Palmer Eye Institute, University of Miami

## Abstract

Uveal melanoma (UM) is the most common primary cancer of the eye and has a strong propensity for metastasis. Although there have been many recent improvements in the diagnosis and treatment of UM, and only 2-4% of patients present with detectable metastasis, up to half of patients are at risk for dying of metastatic disease. Clinicopathologic factors are not accurate enough for individualized patient care. Chromosomal alterations have been used for prognostic purposes, but the routine clinical use of these methods is limited by their susceptibility to sampling error resulting from tumor heterogeneity, limited clinical validation, lack of standardized testing platforms, and high technical failure rates. In contrast, the DecisionDx-UM gene expression profile test is a stand-alone platform which requires no other information for maximal prognostic accuracy and which circumvents many of the drawbacks of chromosomal methods through the use of a highly sensitive microfluidics, PCR-based platform that simultaneously measures the expression of 15 carefully selected genes from primary uveal melanoma samples obtained by fine needle biopsy. Low metastatic risk is reported as Class 1, and high metastatic risk as Class 2. The test allows patients to be stratified into risk categories such that high-risk patients can be offered intensive metastatic surveillance and adjuvant therapy while low-risk patients can be spared these interventions. This test is now used as part of the standard of care in many ocular oncology centers.

## Clinical Scenario

Brachytherapy, proton beam radiotherapy, and enucleation are highly effective treatments for primary uveal melanoma^1^. While only 2-4% of patients have detectable metastatic disease at the time of initial diagnosis, 50% of patients are at risk for fatal metastasis^2^. Traditional staging methods that use clinical and histopathologic prognostic factors, such as the American Joint Committee on Cancer (AJCC) TNM system, can be used to stratify patients into general risk categories^3^, but they do not provide sufficient predictive accuracy to be used for individualized patient care. It has been known for over 20 years that the gain and loss of chromosomal segments is associated with patient outcome, but the study of such chromosomal changes has been most useful as a research tool in large academic medical centers. The use of chromosomal markers in ordinary clinical practice has been hampered by the susceptibility of such tests to sampling error due to intratumoral genetic heterogeneity, limited clinical validation, lack of standardized testing platforms, and high technical failure rates^4^ ^5^ ^6^ ^7^. Further, centers that have attempted to use chromosomal testing platforms for routine clinical use have found that they must include clinical and histopathologic data to improve prognostic accuracy^8^, which introduces technical variability as well as considerable inconvenience for the busy physician in everyday practice.

The DecisionDx-UM^TM^ gene expression profile (GEP) test was designed to address these challenges by providing a highly accurate, stand-alone prognostic test that is straightforward to interpret, requiring no additional information or analysis. The DecisionDx-UM test simultaneously measures the expression of 15 genes from primary uveal melanoma samples obtained by fine needle biopsy in order to determine the likelihood of metastasis. Low metastatic risk is reported as Class 1 whereas high metastatic risk is reported as Class 2. The DecisionDx-UM test allows for implementation of a risk-appropriate metastatic surveillance strategy and accurate identification of high-risk patients who may be eligible for targeted adjuvant therapy prior to the detection of metastasis^9^.

## Test Description

The DecisionDx-UM test uses RNA extracted from a fine needle aspiration biopsy (FNAB) sample, formalin-fixed paraffin embedded (FFPE) post-enucleation specimens, or resected tumor^10^. For fresh FNAB and resected tumor specimens, the sample is immediately submerged into an RNA stabilization buffer and frozen on dry ice. RNA is then converted to cDNA using a reverse transcription kit, cDNA is preamplified for 14 cycles with pooled primers, diluted 20-fold into sterile EDTA buffer and stored at -80°C. RNA expression for the 15 selected genes (12 class discriminating genes and 3 endogenous control genes) is quantified using a real-time PCR system. The number of PCR cycles required for each gene to reach the expression threshold (Ct) is calculated using the manufacturer software, and the mean Ct values are calculated for all sets. The use of pre-amplification and microfluidics ensures a high diagnostic success rate from small FNAB samples.

The assay includes 12 genes that are differentially expressed in Class 1 versus Class 2 tumors (Table 1), and 3 control genes (MRPS21, RBM23, and SAP130) that are expressed at similar levels in Class 1 and Class 2 tumors^10^. The final gene set was selected from a larger number of differentially expressed genes using a rigorous series of filtering steps designed to optimize the performance of the test on scant samples obtained by FNAB^10^. A gene is considered undetectable if its amplification product registers no Ct value after 40 cycles of quantitative PCR, and a sample is considered a technical failure if 1 or more endogenous control genes or at least 3 discriminating genes are undetectable.

The DecisionDx-UM test is currently performed in a single College of American Pathologists (CAP) accredited, Clinical Laborary Improvement Amendments (CLIA) certified laboratory. The test has been performed for over 1500 patients with uveal melanoma in more than 100 ocular oncology centers in North America. Similar to the results recently published for the Collaborative Ocular Oncology Group (COOG) study^11^, technical success for the clinical test, as measured by the number of samples with a reportable Class 1 or Class 2, is over 97%.

**Significance of Class 1 Versus Class 2 Designation**

The DecisionDx-UM test assesses the molecular signature of a patient’s tumor and designates it as Class 1 or Class 2^12^. Class 1 patients have a low risk, and Class 2 patients have a high risk of experiencing a metastatic event in the first five years following diagnosis. In the recent report by the Cooperative Ocular Oncology Group of a prospective multicenter study involving more than 450 patients, >95% of Class 1 patients were free of metastasis at 4 years, compared to <20% of Class 2 patients^11^.

Biologically, the GEP of Class 1 tumors closely resembles that of normal uveal melanocytes and low-grade uveal melanocytic tumors. Conversely, the GEP of Class 2 tumors resembles that of primitive neural/ectodermal stem cells^13^ ^14^.

## Public Health Importance

Uveal melanoma is the most common primary cancer in the eye, with an incidence of 1200-1500 new cases each year^15^. The mortality rate at 15 years of diagnosis of the primary tumor is approximately 50%^2^. Of patients with a Class 2 GEP, approximately half will develop a metastasis within 3 years of diagnosis, and the average survival after metastasis is 9 months^11^ ^16^. Currently there is no effective treatment in preventing deaths from metastatic uveal melanoma; therefore, in order to improve survival, therapies need to be targeted to patients with less advanced disease that is more amenable to treatment. The DecisionDx-UM test allows for implementation of risk-appropriate surveillance plans so that low-risk Class 1 patients receive a low-intensity surveillance plan and high-risk Class 2 patients receive a high-intensity surveillance plan as well as referral to medical oncology for adjuvant therapy opportunities. In addition, the DecisionDx-UM test offers the most accurate method for stratifying patients for inclusion into clinical trials to test the efficacy of adjuvant therapy in high-risk patients^11^.

## PUBLISHED REVIEWS AND RECOMMENDATIONS

**Reviews:** The technique of GEP has been compared to other methods of prognostication for uveal melanoma, including clinicopathologic staging and chromosomal testing, and it has been found to be superior to these other options^17^.

**Professional Organization Reviews:** Gene expression profiling for prognostication is recommended by the American Joint Committee on Cancer (AJCC) in their classification guidelines for uveal melanoma^3^.

**Health Plan/Payer Reviews:** Four external, independent review organization appeals have been completed for “experimental and investigational” and “not medically necessary” payment denials, and all four external appeals concluded that the DecisionDx-UM test was medically necessary and reasonable (Castle Biosciences, Inc., personal communication). Specifics include:

- Independent review request submitted by Blue Cross Blue Shield of North Carolina to the North Carolina Department of Insurance (NCDOI). NCDOI contracted the review to the Medwork Independent Review organization. The appeal concluded that the DecisionDx-UM test was medically necessary.
- Independent review request submitted by Medical Mutual of Ohio. Medical Mutual contracted with National Medical Reviews, Inc. The appeal outcome concluded that the DecisionDx-UM test was medically necessary.
- Independent review request submitted by United Healthcare to Medical Care Ombudsman Program (MCMC). The appeal outcome concluded that the DecisionDx-UM test was not experimental or investigational and that the test did meet covered health service requirements (e.g. medically reasonable and necessary).
- Independent review request submitted by Cigna of Colorado. The appeal outcome concluded that the DecisionDx-UM test was not experimental or investigational.

## EVIDENCE OVERVIEW

**Analytic Validity:**** *Test accuracy and reliability in identifying gene expression profile*

A pubmed search with key words uveal melanoma and gene expression, chromosomal, monosomy 3, staging, or prognosis was conducted in the English literature. The pertinent orginal articles were acquired and reviewed for data and additional references.

The DecisionDx-UM test is a GEP assay that analyzes RNA expression of 15 selected genes, including 3 endogenous controls and 12 class discriminating genes (Table 1). Initially, there was concern that an RNA-based test may not be clinically feasible due to RNA instability. However, a standard operating procedure was developed to rapidly stabilize the RNA immediately after biopsy, which eliminated RNA degradation as a major source of technical failure.

In the DecisionDx-UM test, a gene is considered undetectable if its amplification product registers no Ct value after 40 cycles of quantitative PCR, and a sample is considered a technical failure if 1 or more endogenous control genes or at least 3 discriminating genes are undetectable. A large, prospective multicenter study reported high analytic sensitivity for the test, demonstrating interpretable GEP results in more than 97% of FNAB cases, a higher success rate than that of competing DNA-based tests^11^ ^18^ ^19^ ^20^. This high success rate was in spite of the vast majority of samples being obtained by a single aspiration biopsy using a 25- or 27-gauge needle. A similarly high technical success rate of 97% for class assignment has been observed in a cohort of 1500 uveal melanoma samples that were run in a single CAP accredited, CLIA approved laboratory (Castle Biosciences, Inc., unpublished data).

Technical concordance studies are performed for the DecisionDx-UM test at regular intervals, comparing the classification results obtained in the clinical laboratory to those from the research laboratory. With about 150 samples tested to date, there has been 100% concordance between the laboratories (Castle Biosciences, Inc., unpublished data). Further, the DecisionDx-UM test is regularly evaluated for algorithmic proficiency, which is the ability of the computer algorithm that generates class assignments to render the same result when performed by independent operators. With about 400 samples analyzed to date, there has been 100% concordance between operators (Castle Biosciences, Inc., unpublished data).

Intratumoral heterogeneity for monosomy 3 is estimated to occur in 14-18% of uveal melanomas^7^ ^21^ ^22^; however, heterogeneity for gene expression profiling appears to be significantly lower. In experiments conducted on enucleated eyes, the gene expression profiles of 33 different sections of seven distinct uveal melanomas were studied. For each tumor, sections from the base, apex, and edges were sampled. Only 1 of 33 regions analyzed showed discordance with the other regions within the same tumor. Furthermore, when mixing various ratios of Class 1 and Class 2 tumors, the GEP assay correctly identified all pure Class 1 and Class 2 samples and identified tumors correctly as Class 2 when as little as 25% contribution of Class 2 tumor cells was present in the sample, thus demonstrating that the DecisionDx-UM assay is very sensitive for detecting the Class 2 signature in heterogeneous tumors^10^.

**Clinical Validity:** *Test accuracy and reliability in assigning accurate prognosis in uveal melanoma and comparison to other existing methods*

Retrospective studies from multiple independent institutions have shown that the general technique of GEP is superior in predicting metastasis in patients with uveal melanoma than clinical, pathologic or chromosomal methods^17^ ^19^ ^20^ ^23^. Consistent with these previous studies, the prospective multicenter COOG study found that the specific GEP used in the DecisionDx-UM test was a more accurate prognostic indicator than any other factors tested, including monosomy 3, in patients with uveal melanoma^11^. Other commonly used cytogenetic features, such as gain of 6p and 8q, do not improve upon the accuracy of the DecisionDx-UM test^24^. The authors determined that a likely reason for the superiority of DecisionDx-UM test over cytogenetic methods is that the latter are static markers that are often distributed heterogeneously throughout the tumor and are thus prone to sampling error, whereas DecisionDx-UM test captures a functional “snapshot” of the tumor’s microenvironment that does not vary as much across most tumors and is thus amenable to a single pass of a fine needle in most cases^6^ ^7^ ^10^.

While cytogenetic markers have been widely studied as prognostic factors of uveal melanoma metastasis, the accuracy of methods used to identify cytogenetic abnormalities has been less than sufficient for clinical applicability. For example, the use of fluorescence in situ hybridization for the identification of monosomy 3 in tumors has resulted in as much as a 50% technical failure rate^4^ ^5^. Thus, although cytogenetic studies are indispensable for research purposes, they have not performed as well as the DecisionDx-UM test for routine clinical testing.

The NCCN task force on evalu­ating the clinical utility of tumor markers in oncology stated that the “clinical utility of a marker should be deter­mined in a prospective clinical trial, as is required for new drugs”^25^. Although the NCCN task force has not yet specifically evaluated the DecisionDx-UM test, to date it is the only prognostic test in uveal melanoma that would meet the NCCN criteria for level I evidence for cancer biomarkers, having been validated in a prospective, multi-center study, and it is the only such test that also meets TMUGS criteria for the highest "1a" level of evidence for cancer biomarkers^25^.

**Clinical Utility:**** *net benefit of test in improving health outcomes*

The potential benefits of the DecisionDx-UM test are three-fold. First, the test provides patients with information that most of them desire for personal planning. In two recent studies, 97% of patients reported that they desired to have prognostic information^26^ ^27^, and patients did not report later regret about receiving prognostic information^27^.

Second, the test identifies patients at high risk of metastasis who may benefit from more intensive metastatic surveillance, while sparing low-risk patients of these measures. High intensity surveillance with more frequent and sensitive testing than standard Collaborative Ocular Melanoma Study (COMS) guidelines allows detection and treatment of metastatic disease at an earlier stage, when locoregional treatments such as hepatic chemoembolization and targeted systemic therapies may be more effective^28^ ^29^ ^30^ ^31^.

Third, the DecisionDx-UM test allows accurate stratification to allow entry of high-risk patients into clinical trials to assess the efficacy of adjuvant therapies. Several such trials are already underway, and many more are expected to begin in the coming months as more rationally designed targeted therapies are identified^32^ ^33^.

Clinical use of the DecisionDx-UM test has been recently documented in two independent studies:

(1) Study 1. A blinded survey of ocular oncologists that was presented at the Retina 2012 subspecialty section of the annual meeting of the American Academy of Ophthalmology demonstrated that results of the DecisionDx-UM test impact the follow-up surveillance regimen of patients with uveal melanoma^34^. Seventy-four percent of respondents to that survey indicated that they use the results of the GEP test to “change the frequency of metastatic disease surveillance”. In addition, 23% of respondents refer high risk Class 2 patients to medical oncology for clinical trial consideration.

(2) Study 2. This study involved the systematic review of Medicare cases for 100% of Medicare patients (n=237) who had a reported DecisionDx-UM test result between August, 2010 and January, 2012^35^. Similar to the blinded study noted above, 74% of physicians documented clinical actions that were taken as a result of DecisionDx-UM test results. Specifically, 96% of patients with low-risk Class 1 tumors were placed on a low intensity surveillance plan, whereas 95% of patients with a high-risk Class 2 tumor were placed on a high intensity surveillance plan. Further, 52% of Class 2 patients were provided a medical oncology referral as a result of DecisionDx-UM test results, compared to only 3% of Class 1 patients. Thus, the DecisionDx-UM test allowed patients to be stratified according to risk such that low-risk Class 1 patients were spared the cost and inconvenience of medical oncology referrals and intensive surveillance testing while targeting these resources on the high-risk Class 2 patients who are more likely to benefit from these measures.

## Limitations

The utility of the DecisionDx-UM test has not been assessed for uveal melanomas that have undergone radiation therapy previously. Therefore, collection of the tissue sample must be performed prior to radiation therapy. Also, the test is not recommended for highly necrotic samples.

## Conclusions

The DecisionDx-UM test is the only prognostic tool in uveal melanoma that has been prospectively validated in a multi-center study, and it is used by the majority of ocular oncology centers in North America for routine clinical prognostic testing^36^. The results of the test are used for stratifying patients for risk-appropriate metastatic surveillance, medical oncology referral and entry into clinical trials for adjuvant therapy. The test has been shown to provide superior prognostic accuracy in side-by-side comparisons with clinical, pathologic and chromosomal prognostic factors, and it is simpler to use than these other alternatives. DecisionDx-UM testing on tumor samples obtained from FNAB is much less prone to sampling error due to intratumoral genetic heterogeneity and technical failure due to insufficient tumor material compared to chromosome-based tests^10^. As targeted therapies for metastatic uveal melanoma are rapidly emerging, the DecisionDx-UM test allows appropriately selected high risk patients with Class 2 tumors to be selected for clinical trials, thereby reducing both the number of patients required and the amount of time needed to achieve appropriately powered clinical trials designed to detect differences in outcomes.

## Table 1

**Discriminating genes comprising the DecisionDx-** UM^TM^ **gene expression profile prognostic test for uveal melanoma**

**Table:** 

**Gene Symbol**	**Gene Name**	**Direction of Change in Class 2 Tumors**
*CDH1*	E-cadherin	Up
*ECM1*	Extracellular matrix protein 1	Up
*E1F1B*	Eukaryotic translation initiation factor 1B	Down
*FXR1*	Fragile X mental retardation autosomal homolog 1	Down
*HTR2B*	5-hydroxytryptamine (serotonin) receptor 2B	Up
*ID2*	Inhibitor of DNA binding 2	Down
*LMCD1*	LIM and cysteine-rich domains 1	Down
*LTA4H*	Leukotriene A4 hydrolase	Down
*MTUS1*	Microtubule-associated tumor suppressor 1	Down
*RAB31*	RAB31, member RAS oncogene family	Up
*ROBO1*	Roundabout, axon guidance receptor 1	Down
*SATB1*	SATB homeobox 1	Down

## Competing Interests:

Dr. Harbour is the inventor of intellectual property used in the study and receives royalties from its commercialization. He is a paid consultant for Castle Biosciences, licensee of intellectual property presented in this article.
